# Supplementary material for: Controlling toxic and harmful gas in blasting with an inhibitor
Source: PLoS One. 2023 Dec 18;18(12):e0291731. doi: 10.1371/journal.pone.0291731 (PMC10727359; doi:10.1371/journal.pone.0291731)
Supplement: Attachment — Submitted filename: comments.docx [file pone.0291731.s001.docx]

Zhang Xiliang et. al. reported the Controlling toxic and harmful gas in blasting with an inhibitor. The authors have developed an efficient blasting harmful gas inhibitor based on the analysis of the mechanism of the inhibitor's action. The appropriate addition ratio and charging structure were determined through experiments, achieving good on-site test results and providing an effective technical solution for controlling harmful gases in mining blasting. However, a few more clarifications are needed before the manuscript can be considered suitable for publication. A list of other comments that need to be addressed is as follows:

1. Author should discuss more about role of Inhibitor.

2. Amount of charge in a hole and average crack rate should be explained in result and discussion part.

3. Comparison of present study to literature should be included.

4. In the introduction section, the recent development of sensors towards toxic gases like NO, NO_2_, which can help to provide a full background to the readers, to provoke the readers’ new ideas, and to improv the impact of the present job, some key publications such as Materials science and engineering B, 247, 2019,114381, Ceramic International,44, 2018, 4392, Applied Surface Science, 396 (2017) 1317.

5. Some errors and inappropriate places should be corrected, e.g. such as ---etc; NH3 (line 72) should be NH_3_.

6. In table 2 and table 4, the gases should be in English language.
