# Supplementary material for: Controlling toxic and harmful gas in blasting with an inhibitor
Source: PLoS One. 2023 Dec 18;18(12):e0291731. doi: 10.1371/journal.pone.0291731 (PMC10727359; doi:10.1371/journal.pone.0291731)
Supplement: Attachment — Submitted filename: Response to Reviewers.docx [file pone.0291731.s002.docx]

**Dear Editors and Reviewers:**

Thank you for your letter and for the reviewers’ comments concerning our manuscript entitled “***Controlling toxic and harmful gas in blasting with an inhibitor***” (ID: PONE-D-23-20758). Those comments are all valuable and very helpful for revising and improving our paper, as well as the important guiding significance to our researches. We have studied comments carefully and have made correction which we hope meet with approval. Revised portion are marked in red in the paper. The main corrections in the paper and the responds to the reviewer’s comments are as flowing:

**Responds to the reviewer’s comments (the changes marked in red in revised paper):**

# Reviewer #1:

1. Comment: Author should discuss more about role of Inhibitor.
**Response:** The author add more information on the effects of inhibitors.

Page 2-3, Line 83-137

2. Comment:Amount of charge in a hole and average crack rate should be explained in result and discussion part.
**Response:** The author explain the amount of charge in a hole and average crack rate in result and discussion part(3).

Line 471-474

3. Comparison of present study to literature should be included.
**Response:**The author add more information about comparison of present study to literature in result and discussion part(2).

Line 457-460

4. Comment: In the introduction section, the recent development of sensors towards toxic gases like NO, NO_2_, which can help to provide a full background to the readers, to provoke the readers’ new ideas, and to improv the impact of the present job, some key publications such as Materials science and engineering B, 247, 2019,114381, Ceramic International,44, 2018, 4392, Applied Surface Science, 396 (2017) 1317.
**Response:** The author added these three references in the introduction section.

Line 63-67

Line 518-424

5. Comment: Some errors and inappropriate places should be corrected, e.g. such as ---etc; NH_3_ (line 72) should be NH_3_.

**Response:** The author has made modifications, see red font.

Line 94

6.Comment: In table 2 and table 4, the gases should be in English language.

**Response:** The gas is expressed in English in table 2 and table 4, see red font.

Line 200	288

# **Reviewer #2:**

1. Comment: Line 141, author states "From Table 2, Figure 6, and Figure 7, it can be seen that with the increase of the proportion of blasting harmful gas inhibitors added, the concentration of toxic and harmful gases shows a trend of first decreasing and then increasing, and the gas concentration reduction rate shows a pattern of first increasing and then decreasing." Why is this trend followed is not clearly mentioned by the author.

**Response:** The author analyzes and believes that different inhibitor addition ratios will produce different control effects on harmful gases during blasting, and only when the addition ratio is appropriate can the best control effect be achieved.

Line 207-209

2. Comment: In section 4, author should show the superiority of their work by referencing or mentioning about the effect of other factors such as humidity, temperature, and pressure in their proposed solution.
**Response:** The temperature of the test working surface is 38~32℃, the humidity is 80%~86%, and the pressure is one atmospheric pressure.

Line 239-241

3. Comment: Add references for the statement in Line 230.
**Response:** Reference 19-20 have been added here.

Line 303

4. Comment: In section 3, line 307, author should mention why their data for CO concentration vs distance is deviating from the trend as observed in NH_3_ and other gases concentration comparison.

**Response:** It should be noted that the variation pattern of CO concentration with distance is generally consistent with that of NH_3_. There is a certain small fluctuation at local monitoring points, and analysis suggests that it is a normal phenomenon due to the non uniform diffusion process of CO, which is affected by wind and other factors.

Line 402-405

# **Reviewer #3:**

1. Comment:Author didnt provide details of harmful gas inhibitors (A, B, D). What are these elements ? Is the inhibitor reaction with harmful gases thermodynamically feasible ? Enthalpy of reaction discussion missing ?
**Response:** A is the element Silicon; B is the element Iron; D is the element Copper. Since no patent has been applied for protection, the letter A、B、D are used for representation.

The reaction between inhibitors and harmful gases is thermodynamically feasible.

2. Comment: Line 51 - Full form of CFD ?
**Response:** The full name of CFD is Computational Fluid Dynamics, which is a software of Fluent.

Line 53

3. Comment: Line 102 - Short description of Fig 1 in the text to explain the action principal of inhibitors and harmful gas . Is there any significance of grouping the gases in specific colors ?
**Response:** As shown in Figure 1, different colors represent different chemical reaction processes, corresponding to the chemical reactions between the explosive toxic gases NO_2_, NO, CO, NH_3_ and the inhibitor, achieving the control of these four harmful gases.

Line 135-137

4. Comment: Line 114 - More information required in the text with details
Fig3 - Details of the X-am detector
Fig4 - What chemicals are these ? How they were prepared ?
Fig5 - Is it detonator and explosives ? If yes then the details are required. The image is not very clear. Recommedn to add magnified image of the white thing with explaination what is it ?
**Response:** Fig3 - Details of the X-am detector. The X-am5000 portable composite gas detector can effectively measure the concentration of harmful substances such as CO, H_2_S, CO_2_, NH_3_, NO_2_, and SO_2_. Size: (length × wide × Height) 147mm × 129mm × 31mm; Weight: 220 grams; Environmental conditions: Temperature: -20~+50℃; Pressure: 700~1300hPa; Humidity: 10%~95% RH.

Fig4 -On the left is emulsion explosive. On the right is a mixture of explosives and inhibitors. They are formed by stirring and mixing evenly with a glass rod.

Fig5 -Above is the detonator.

Below is a mixture of explosives and inhibitors, wrapped in a white paper.

Line 155-162

Fig. 4 Experimental materials	Fig. 5 Experimental photos

5. Comment: Line 120 - Which technique was used to collect the concentration ?
**Response:** The X-am detector was used to collect the concentration as mentioned above.

6. Comment: Line 130 - What temperature value ? Reference required if lab data is not available
**Response:** Under normal temperature conditions, 20~25℃.

Line 191

7. Comment: Line 138 - Please explain the character used as table heading ?
**Response:** Carbon monoxide (CO); Nitric oxide (NO); Nitrogen dioxide (NO_2_)

Table 2 Inhibitor addition test data table

| Add scale | Carbon monoxide (CO) | | Nitric oxide (NO) | | Nitrogen dioxide (NO_2_) | | Total | |
| --- | --- | --- | --- | --- | --- | --- | --- | --- |
|  |  |  |  |  |  |  |  |  |

Line 200

8. Comment: Line 269 - Why Scheme 3 has just 3 points ? How ppm/kg numbers are calculated for different schemes ?
**Response:** It should be noted that Scheme 3 was used to monitor the gas concentration at three different distance points, and the monitoring data was significantly higher than other schemes. It can be determined that this scheme has poor performance, so no further monitoring was conducted at other distances. Therefore, only three measurement point data were used.

The gas concentration monitored by the X-am detector is in ppm, which is divided by the amount of charge used (in kg) to obtain ppm/kg.

Line 340-344

9. Comment: Line 315 - Why Scheme 1 slope is different compared to scheme 2 ? What does the slope of the curve signifies and what factors will influence it.

**Response:** During the diffusion process of harmful gas from blasting, it is influenced by factors such as the shape of the tunnel contour, changes in air flow, and gas density. The concentration of different gases may fluctuate in a small range, but it does not affect the consistency of the overall trend, making Scheme 1 slope different compared to Scheme 2.

The slope of the curve is influenced by factors such as the shape of the tunnel, changes in air flow, and gas density. It only represents the variation of gas concentration with distance and reflects the attenuation process of gas concentration values.

# **Reviewer #4:**

1. Comment: Introduction: While your review of previous works is comprehensive, it lacks clarity on the novelty and differentiation of your study. Please explicitly state how your work differs from previous research, particularly regarding the use of harmful gas inhibition. (Line 30-66)
**Response:** Compared with previous research, the blasting harmful gas inhibitor developed in this article is a mixture of multiple elemental substances, oxides, steady-state agents, and water, with more complex components. It overcomes the shortcomings and shortcomings of previous inhibitors with single components and less significant harmful gas control effects, and can achieve more obvious harmful gas suppression effects.

Line 72-76

2. Comment: Composition of A, B, D elements: Please provide a clear explanation of the composition for elements A, B, and D, as mentioned in the paper. This information is essential for readers to understand the materials used. (Line 69)

**Response:** A is the element Silicon; B is the element Iron; D is the element Copper.

Since no patent has been applied for protection, the letter A、B、D are used for representation.

3. Comment: Stabilizing Agent: Clarify the specific organic and environmentally friendly stabilizing agent used and elaborate on how it ensures efficient mixing. (Line 70)
**Response:** The stabilizing agent here can promote the uniform dispersion of inhibitor particles in the medium, including types such as water glass, cellulose derivatives, polyacrylamide, and gum. During the experiment, the stabilizing agent, inhibitor, water, and explosives were placed together in a plastic cup, and a glass rod was used to continuously stir until the stirring was uniform, ensuring effective mixing of various substances.

Line 83-87

4. Comment: Reaction Conditions: Include the temperature and pressure conditions at which the inhibitor undergoes the reaction, as this information is crucial for understanding the kinetics of the process. (Line 71)
**Response:** Explosive explosions produce high-temperature and high-pressure gases at the moment. Explosion temperature refers to the heat released during the explosion of an explosive, which heats the explosive product to the highest temperature. Different types of explosives have different explosion temperature ranges, usually between 2000~3000 ℃. Detonation pressure is the pressure on the detonation front of an explosive during explosion, and its value mainly depends on the chemical properties of the explosive, usually ranging from 10 to 40GPa.

Line 88-93

5. Comment: Mechanism of Harmful Gas Inhibitors: In the section explaining the mechanism of harmful gas inhibitors in blasting, address whether additional reactions, such as NH3+N2+H2 → NH4NO3 (or any other byproducts), might be generated. If so, provide information on the toxicity of these byproducts. (Line 67)
**Response:** Of course, due to the instantaneous and unmeasurable nature of explosive explosion reactions, there may also be additional chemical reactions, such as NH_3_+N_2_+H_2_ → NH_4_NO_3_. Due to the lack of effective monitoring instruments for detecting the explosion process, the author believes that there is a certain possibility of this reaction. NH_4_NO_3_ is highly soluble in water, prone to moisture absorption and agglomeration, and can decompose explosively under strong impact or heat. It is mainly used as fertilizer, industrial and military explosives. It has a certain degree of irritation to the eyes, respiratory system, and skin of the human body. Due to the unstable heating characteristics of NH_4_NO_3_, it is believed that NH_4_NO_3_ will not exist in the final product of the explosion and may only exist as a product of the explosion process.

Line 138-146

6. Comment: Dimensions of Closed Container: Clarify the dimensions of the closed container used for the blasting experiment in line 105.

**Response:** long × wide × Height=1m × 1m × 1.5m.

Line 151-152

7. Comment: Control Experiments: Explain whether all control experiments are identical. If so, please address the discrepancy in Table 1, where the ppm for NO generated in the 3rd test is approximately 33% higher than the average of test 1 and 2. Clarify whether this deviation is expected or requires further investigation. (Line 117)
**Response:** The production process of emulsion explosives adopts a large-scale production process, and various components of the explosives inevitably have certain non-uniformity during the stirring preparation process, which has a certain impact on the performance of the explosives and the production of harmful gases. The different properties of explosives, the varying degrees of response to explosive explosions, and the different usage environments can all have a certain impact on the production of toxic and harmful gases.

It was precisely considering these factors that the author conducted three experiments, taking the average of the three experiments to overcome the impact of uneven explosives and unstable performance on the experimental results. The high concentration of NO in the third test is consistent with the uneven distribution of explosives. After averaging the three test zones, this effect can be eliminated overall.

Line 177-186

8. Comment: Table 2: Provide clarification for the Chinese character in Table 2.
**Response:** The Chinese character is Total.

We are very sorry for our incorrect writing.

Line 200

Table 2 Inhibitor addition test data table

| Add scale | Carbon monoxide (CO) | | Nitric oxide (NO) | | Nitrogen dioxide (NO_2_) | | Total | |
| --- | --- | --- | --- | --- | --- | --- | --- | --- |
|  |  |  |  |  |  |  |  |  |

9. Comment: Saturation at 6%: In lines 153-155, explain why the performance saturated at 6%
**Response:** There is a certain proportional relationship between the amount of inhibitor added and the control effect of harmful gases during blasting, and it is not advisable to add too little or too much. When the amount of inhibitor added is too small, the harmful gas control effect cannot be achieved, and when too much is added, it can cause waste of inhibitor.

According to the experiment, it can be found that there is a significant turning point when the inhibitor addition ratio is 4%, at which point the maximum reduction in harmful gases is achieved. Therefore, when the inhibitor addition ratio is greater than 6%, it can be considered that it will cause waste of inhibitors, which is uneconomical.

Line 220-227

10. Comment: Inhibition Effect Between 4% and 6%: Based on the data, 4% showed the highest reduction in harmful gas concentration. Have you explored the inhibition effect between 4% and 6%? If so, please include those findings.

**Response:** The author did not conduct research on the addition of inhibitors between 4% and 6%. We think this is a very good suggestion.

This will be the author's next research work to further optimize the inhibitor addition scheme and achieve the optimal addition ratio.

11. Comment: Onsite Testing: For the different schemes of onsite testing, specify the amount of inhibitor used. Was it 4% of the emulsion explosive?
**Response:** Yes, it was 4% of the emulsion explosive.

12. Comment: Quantity of Emulsion Explosive Used: In line 169, share the quantity of emulsion explosive used for the onsite testing.
**Response:** Due to different on-site production conditions, the amount of explosives used for each blasting varies, ranging from 11.1 to 156kg, as shown in Fig.12-13.

Line 3220-324

Fig 12 Explosive usage for different schemes

Fig 13 Toxic and harmful gases generated by different blasting schemes

13. Comment: Scheme 1 Performance: Explain why Scheme 1 performed better than other schemes in the onsite testing. Provide a more comprehensive conclusion to elucidate the mechanism behind why 4% and Scheme 1 yielded superior results.
**Response:** The contact area between inhibitors and explosives is one of the key factors affecting the control effect of harmful gases in blasting. The larger the contact area between the two, the more sufficient the reaction between the two, and the better the control effect of harmful gases. In Scheme 1, the contact area between the inhibitor and the explosive is the largest, which is greater than the other two schemes, so Scheme 1 performs the best.

There is a certain proportional relationship between the amount of inhibitor added and the control effect of harmful gases during blasting, and it is not advisable to add too little or too much. When the amount of inhibitor added is too small, the harmful gas control effect cannot be achieved, and when too much is added, it can cause waste of inhibitor.According to the experiment, it can be found that there is a significant turning point when the inhibitor addition ratio is 4%, at which point the maximum reduction in harmful gases is achieved. Therefore, it can be considered that adding 4% has the best effect.

We tried our best to improve the manuscript and made some changes in the manuscript. These changes will not influence the content and framework of the paper. And here we did not list the changes but marked in red in revised paper.

We appreciate for Editors/Reviewers’ warm work earnestly, and hope that the correction will meet with approval.

Once again, thank you very much for your comments and suggestions.

Thank you and best regards.
Yours sincerely,
Haibao Yi

**Corresponding author:**

Name: Haibao Yi
E-mail:hang_tianfeji@126.com
