# Supplementary material for: Controlling toxic and harmful gas in blasting with an inhibitor
Source: PLoS One. 2023 Dec 18;18(12):e0291731. doi: 10.1371/journal.pone.0291731 (PMC10727359; doi:10.1371/journal.pone.0291731)
Supplement: Attachment — Submitted filename: comments 1.docx [file pone.0291731.s003.docx]

Conclusion on research article:

Zhang Xiliang et. al. reported the Controlling toxic and harmful gas in blasting with an inhibitor. All the comments are addressed properly, that manuscript is ready for publication.
